# Effects of 17α-methyltestosterone and letrozole on growth and gonadal development in largemouth bass (*Micropterus salmodies*)

**DOI:** 10.3389/fphys.2024.1444918

**Published:** 2024-09-17

**Authors:** Dongyun Zhang, Shengjie Li, Taihang Tian, Jinxing Du, Caixia Lei, Tao Zhu, Linqiang Han, Hongmei Song

**Affiliations:** ^1^ Pearl River Fisheries Research Institute, Chinese Academy of Fishery Sciences, Key Laboratory of Tropical and Subtropical Fishery Resource Application and Cultivation, Ministry of Agriculture and Rural Affairs, Guangzhou, China; ^2^ Guangdong Provincial Key Laboratory of Aquatic Animal Immunology and Sustainable Aquaculture, Pearl River Fisheries Research Institute, Chinese Academy of Fishery Sciences, Guangzhou, China; ^3^ College of Life Science, Huzhou University, Huzhou, China; ^4^ College Fisheries Science Education, Shanghai Ocean University, Shanghai, China; ^5^ Guangdong Province Liangshi Aquaculture Seed Industry, Foshan, China

**Keywords:** MT, LE, growth, gonadal development, all-female largemouth bass

## Abstract

In order to optimize the parameters for reversing masculinization and establish the techniques for sex induction of pseudo-males and creation of all-female fry in largemouth bass (*Micropterus salmodies*, LMB), 15-day-old LMB (1.00 ± 0.10 cm in length, 0.10 ± 0.01 g in weight) were fed a diet supplemented with either 17α-methyltestosterone (MT) or letrozole (LE) and their combination. The experimental groups were M20 (20 mg/kg MT), L20 (20 mg/kg LE) and M10L10 (10 mg/kg MT and 10 mg/kg LE). The control group, named C, was not feed MT or LE. After 60 days, exogenous hormone in the diets was stopped and the effects of MT and LE on growth, male ratio, and gonadal development in LMB were evaluated. At 12-month-old, blood and gonadal tissue samples were collected to measure sex steroid hormones levels, analyze expression levels of *dmrt1* and *cyp19a1a* genes, as well as examine the gonads morphology. The results showed no significant differences in growth between the experimental groups and the control group after a 60-day feeding period with the formulated diet (*p* > 0.05). The sex reversal ratio of M20, L20, M10L10 were 95.00%, 80.00%, 76.47%, respectively. The gonadal tissue sections showed that the gonadal structure of masculinized fish morphologic resembled that of control male fish. At 12-month-old, the sex reversal ratio in M20, L20, M10L10 and C groups were 100%, 86.67%, 73.33% and 50.00%, respectively. The testicular of pseudo-male fish in the M20 group exhibited well-developed morphology similarities to that of the control group males. However, the testes of pseudo-male fish in the L20 and M10L10 groups were smaller size Estradiol (E_2_) levels in the experimental groups was significantly lower than those in the control group females (*p* < 0.05), while testosterone (T) levels were significantly higher than that of the control group (*p* < 0.05). Compared to the female fish in the control group, pseudo-male fish from all experimental groups showed significantly upregulated expression of *dmrt1* (*p* < 0.05), and significantly downregulated expression of *cyp19a1a* (*p* < 0.05). Pseudo-males selected from group M20 exhibited a significantly higher proportion of female offspring (92.00%) compared to the control group (46.50%). In summary, 20 mg/kg MT was the optimal inducing concentration.

## Introduction

The largemouth bass (*Micropterus salomides*, LMB), commonly known as the California perch, belongs to the Perciformes order, Percoidei suborder, Centrarchidae family, and *Micropterus* genus. It is naturally distributed in freshwater habitats of North America and was introduced to China in 1983. Due to its delicious and tender flesh without intermuscular spines, rendering it ideal for primary and deep processing by consumers (Bai and Li, 2018), the LMB has become a popular species for freshwater aquaculture in China. Through extensive promotion across 29 provinces and cities, its production has reached an impressive 802,000 tons in 2022. It has been identified that female largemouth bass grow faster and have larger body size than male largemouth bass in the 1-year cultivation model (Arslan T, 2002; Wei, 2022). Therefore, breeding whole female LMB is advantageous for reducing the cost and increasing the efficiency of breeding. In preliminary experiments, although Arslan and Porter et al. (2010), Porter et al., 1996 successfully increased the male ratio by feeding 17α-methyltestosterone (MT), they did not monitor the reproductive capacity of pseudomorphs during the breeding period. In our previous study, type of sex determination in largemouth bass has been confirmed to be XX/XY, and we have successfully induced pseudo-males in LMB using MT at doses of 50–100 mg/kg (Zhou et al., 2021). Moreover, previous histological section results showed that from 20 dph to 30 dph, the germ cells gradually differentiated into oogonium and spermatic deferent, respectively (Du, et al., 2022). However, this resulted in testicular hypoplasia and reproductive dysfunction, subsequently reducing the reproductive efficiency and breeding value of pseudo-males.

In juvenile fish, the determination and differentiation of fish sex can be easily influenced by exogenous hormones, such as MT. It has been found that MT can induce the production of pseudo-male fish (Sualeman and Fotedar, 2017). When precisely administrated, MT acted similarly to endogenous androgens, facilitating testicular development. However, higher concentrations of MT have been shown to hinder testes development (Zhang, 2010) and potentially lead to hypoplasia of the reproductive system (Josefa et al., 2011). At the dose of 50 and100 mg/kg MT, positive correlation between male ratio and concentration of MT was observed, up to the concentration of 200 and 250 mg/kg, thereafter reproductive failure was noticed in green terror (*Aequidens rivulatus*) (Wang et al., 2023) After being exposed to MT (25, 50, and 100 ng/L) in male stone moroko (*Pseudorasbora parva*), the inhibitory effect of MT was gradually increased in male spermatogenesis, and the number of mature spermatozoa was subsequently decreased (Liu et al., 2016). Feeding topmost culter (*Culter alburnus*) with diets containing 6 and 60 mg/kg MT, respectively, showed that the concentration group of 60 mg/kg exhibited a more obvious inhibitory effect on testes development (Wang et al., 2018). During the process of sexual reversal, higher concentrations of MT were found to impede testes development, while lower concentrations of MT treatment could reduce reproductive functioning in pseudo-male fish (Devlin and Nagahama, 2002). The fecundity of pseudo-male three spotted tilapia (*Oreochromis Andersonii*) fed a diet containing 40 mg/kg MT was significantly higher than that of the experimental group with 60 mg/kg MT, and showed no significant difference compared to the control group (Kefi et al., 2012). Mercedes et al. (1995) supplemented the diet with 10 mg/kg of MT, which resulted in the successful inducing reproductive ability in pseudo-male sea bass (*Dicentrarchus labrax)*. Similarly, pseudo-male large-scale salmon (*Oncorhynchus tshawytscha*) were obtained by using 400 μg/L MT and effectively harvested the all-female population though pairing them with normal female fish (Hunter et al., 1983).

Following the ingestion of MT, cultured fish exhibit a relative increase in androgen levels accompanied by a comparative decrease in estrogen levels, thereby inducing masculinization of female fish (Chi et al., 2019). Aromatase inhibitors with similar functionality, such as letrozole (LE), competitively bind to aromatase, reducing estrogen production and inhibiting ovarian development while promoting gonads differentiation into testes (Buzdar et al., 2002). The embedding of 5 mg/kg LE can result in obtaining 50% of fertile red spotted grouper (*Epinephelus akaara*) (Li et al., 2005). When different doses of LE (20, 50, 150 and 300 mg/kg) were added to the diet, the male proportion of yellow catfish (*Pelteobagrus fulvidraco*) increased with higher concentrations. The testes tissue structure of pseudo-male fish showed no significant difference compared to that of the control group (Shen et al., 2013). Feeding rainbow trout (*Oncorhynchus mykiss*) a diet containing 50 mg/kg of LE resulted in slow spermatogonia development in pseudo-male rainbow trout, but eventually led to the maturation and formation of complete testes (Xu et al., 2021). The addition of 10 mg/kg LE to the diet resulted in 100% masculinization of female striped rock bream (*Oplegnathus fasciatus*), and the testes of pseudo-males were filled with spermatocytes and sperms (Liu et al., 2023). Furthermore, simultaneous addition of 5 mg/kg MT and 300 mg/kg LE to the feed for yellow catfish resulted in a testis tissue structure similar to that of the control group (Liu, 2022).

In order to further optimize the technique of pseudo-male LMB, we used lower concentrations of MT and LE. In this study, we evaluated the effect on growth, counted the proportion of males, observed the structure of gonadal tissues, analyzed the level of steroid hormone and detected the expression of *dmrt1* and *cyp19a1a*. These findings will contribute towards optimizing masculinization induction protocols for LMB and provide technical insights for parthenogenetic breeding of this species.

## Materials and methods

### Preparation of experimental feed and feeding management

The experimental juvenile LMB of was raised by circulating aquaculture system at Liangshi Aquaculture Seed Industry, Foshan, Guangdong. MT (MCE#HY-A0121) and LE (MCE#CGS20267) were purchased from Guangzhou Weijia Technology Co., Ltd. LMB. The diameter of the circular aquaculture tank is 1 m, the water depth is also 1 m. Each tank (1 m^3^) contained 1000 LMB (15-day-old, 1.00 ± 0.1 cm in length, 0.1 ± 0.01 g in weight), and initially composed of potential males and females (room temperature, natural light). The experimental groups were fed with 20 mg/kg MT, 20 mg/kg LE, and 10 mg/kg MT+10 mg/kg LE, named M20, L20, and M10L10, respectively. Control group feed did not contain MT or LE, named C, females and males in the C group named C-F and C-M, respectively. Each group is arranged with triplicate, a total of 12 in number. The MT and LE was dissolved in anhydrous ethanol and methyl sulfoxide to prepare a solution of 5 mg/mL, respectively. and stored at 4°C. Based on the experimental concentrations, the required solution volumes were calculated, and diluted the solution to spray them on the diets according to the method of El-Greisy and El-Gamal, 2012. All the diets were placed in a cool and dry place to air dry, and stored at 4°C. The 20 mg/kg MT or 20 mg/kg LE were alone sprayed in the diets, the group of M10L10 (10 mg/kg MT+10 mg/kg LE) was mixed in the diet. The control group was sprayed with anhydrous ethanol. The feeding duration for MT and LE groups lasted for 60 days, during which they were provided with satiation three times a day at 8:00, 12:00 and 17:00. Residual bait and stain were cleaned up after each meal. Subsequently, all experimental fish and the control group were tagged with bio-glass tube tags (RBC-B1.4*8) and transferred to the cement pools (7 m × 3 m × 1.2 m), where they were provided with the same diets as the control group until reaching 12-month-old.

### Sampling and analysis

After 30 and 60 days of administration, 30 LMB were randomly selected from each group to measure their body length (L) and weight (W), followed by calculating specific growth rates for both parameters. Additionally, after a 60-day administration period, another set of 30 LMB were randomly selected from each group. Caudal fins were preserved in ethanol for subsequent genetic sex determination (Du et al., 2021), while gonads were fixed in a 4% paraformaldehyde solution for histological examination using hematoxylin-eosin staining (HE). At 12 months old, 30 fish were randomly selected from each group for collecting caudal fins, blood samples, and gonads. Caudal fins were also used for genetic sex determination. Blood samples were centrifuged at 3000 rpm at 4°C for 15 min and serum was stored at −80°C for analysis of estradiol (E_2_) and testosterone (T) concentrations. The portion of dissected gonads was preserved at −80°C to assess the gene expression levels of *dmrt1* and c*yp19a1a*. Another portion was fixed in 4% paraformaldehyde for HE sections. After MT and LE treatment, the experimental fish in each group were mixed cultured with electronic chips implanted in their abdomen, meanwhile, their tail fins were collected for sex identification.

Specific growth rates for body length and body weight are calculated as follows:

Specific growth rate of body length: SGR_L_ (%) = 100 × (lnL_2_-lnL_1_)/t, Specific growth rate of body weight: SGR_W_ (%) = 100 × (lnW_2_-lnW_1_)/t.

L_1_: initial body length (cm); L_2_: final body length (cm); W_1_: initial body weight (g); W_2_: final body weight (g); t:30d.

### Paired breeding of 12-month-old pseudo-male LMB

At 12-month-old, pseudo-males with genotype XX were first screened by electronic labeling, and then pseudo-males that could extrudate sperm were selected. Finally, 12 well-developed pseudo-males were selected in group M20. Normal female fish and pseudo-male fish were placed in an indoor cement pool with an area of 21 m^2^ for pair breeding using Li’s artificial breeding method (Li, 2008), referred to as the all-female group. Simultaneously, 12 pairs of male and female fish with mature gonads that were selected from the control group for reproduction. The fertilized eggs were transferred to the incubation tank separately for incubation. The gonads of randomly sampled 200 LMB from both the all-female group and the control group at 120 days of age were dissected to determine their sex and calculate the proportion of females.

### Preparation of HE tissues sections

The gonad tissue samples were dehydrated with gradient ethanol, transparentized in benzyl alcohol, embedded in paraffin, and sectioned in 5–8 μm. Then the sections were stained with hematoxylin-eosin (HE) and observed and photographed with ZEISS microscope (Axio Scope. A1). And the type of gonads and cells were determined according to the method of Liu and Rhody et al. (Liu, 1993; Rhody et al., 2013).

### Detection of serum E_2_ and T concentrations

The gender markers of LMB were used to identify pseudo-males in the experimental group, as well as females and males within group C. Specifically, females in group C were referred to as the C-F group, while males in group C were referred to as the C-M group. E_2_ and T levels were detected following the instructions provided by the Fish Estradiol (E_2_) and Testosterone (T) ELISA Kit (Shanghai Sinobestbio Biotechnology Co., Ltd.). The optical density (OD) was determined at 450 nm with a spectrophotometer (Biotek Cytation5, United States of America). Subsequently, the concentrations of E_2_ and T in the samples were determined by comparing their O.D. values to the standard curve.

### RNA extraction and gene expression

Total gonadal RNA was extracted from pseudo-male LMB in experimental groups and the control group using Tissue RNA Kit (OMEGA). The purity and concentration were detected with 1% agarose gel electrophoresis and multifunctional enzyme labeler (Biotek Cytation5, Winooski, VT, United States of America), respectively. The cDNA synthesis was performed with reference to the Takara reverse transcription kit. While qRT-PCR analysis was performed using the Thermo Feld fluorescence quantifier (QuantStudio 6). The reaction system of qRT-PCR was: cDNA 2 μL, forward primer 0.5 μL, reverse primer 0.5 μL, Premix 10 μL, DEPC H2O 7 μL, 20 μL in total. The reaction procedure was: 50°C 2 min, 95°C 10 min, (95°C 15 s, 60°C 30 s, 72°C 30 s, 40 cycles), 95°C 15 s, 60°C 1 min, 95°C 15 s. Using *β-actin* as reference (Zhou, 2021), the relative expression levels of *dmrt1* and *cyp19a1a* genes were detected. The reaction system of PCR of sex determination was: DNA1 μL, forward primer 1 μL, reverse primer 1 μL, Premix 10 μL, DEPC H2O 7 μL, 20 μL in total. The reaction procedure was: 95°C 3 min, 95°C30 s, 56°C 30 s, 72°C 1 min, 72°C10 min, 35 cycles. The primer was synthesized by Guangzhou IGE Biotechnology Co., Ltd. (Table 1). The relative expression was calculated by 2^-△△Ct^ method (Livak K J and Schmittgen T D, 2001).

**TABLE 1:** Nucleotide sequences of real-time PCR primers.

Gene/primer name	Forward primer	Reverse primer
*dmrt1*	GCT​CCC​GCT​GTA​GGA​ACC​AC	CCT​GAG​CCT​GCT​GCC​TTC​TC
*cyp19a1a*	GTG​AGG​CAG​TGT​GTG​CTG​GA	CAG​CCG​CAG​CTC​CAC​ATC​T
*β-actin*	AAA​GGG​AAA​TCG​TGC​GTG​AC	AAG​GAA​GGC​TGG​AAG​AGG​G
sex marker	CCG​GAG​CTA​ACC​TCT​GTT​GC	CGG​GGC​AAC​CTG​CAA​GAT​TA

### Data statistics and analysis

The experimental data was sorted by Excel 2016 Microsoft, and the data analysis was analyzed using SPSS 20 software for One-Way ANOVA and multiple comparison (Duncan) analysis 0.05 was a significant difference. The test data is represented by mean plus or minus standard deviation.

## Results

### Effects of MT and LE feeding on the growth of LMB

**FIGURE 1:**
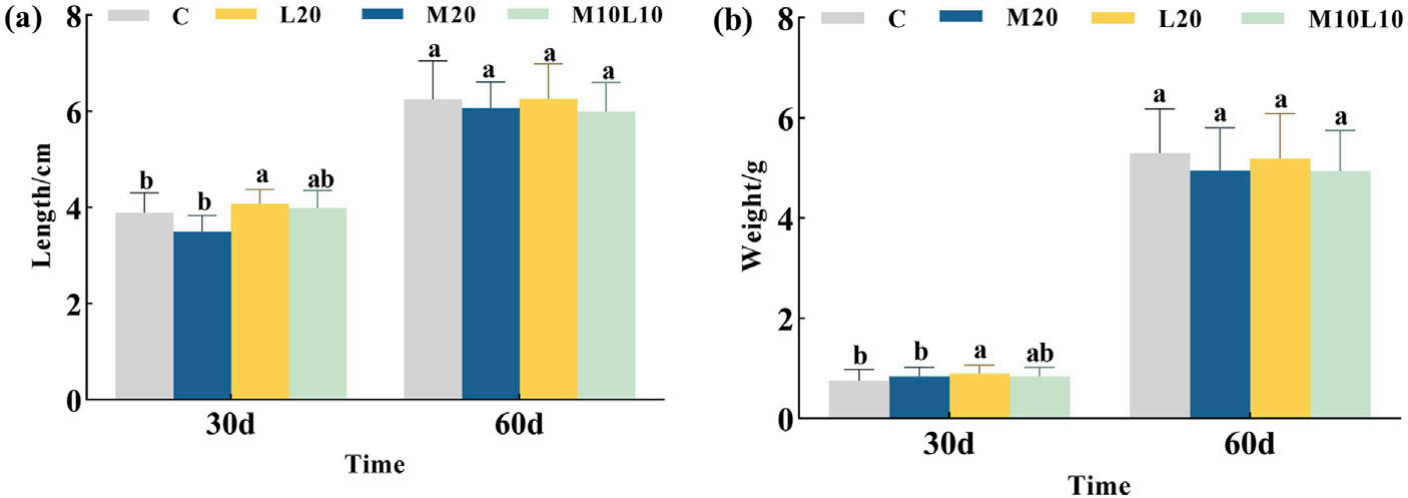
Effects of MT and LE on body length **(A)** and body weight **(B)** of LMB. Note: Different letters between the treatments in the same period show significant difference (*p* < 0.05), the same letter indicates that the difference is not significant (*p* > 0.05).

After being fed diets containing MT and LE for 30 days, the body length and weight of the M20 group did not differ significantly from those of the C group (*p* > 0.05), while the L20 group showed significantly higher body length and weight compared to the C group (*p* < 0.05). However, after a feeding of 60 days, there were no statistically significant differences observed in terms of body length and weight between the experimental groups and the control group (Figure 1). The statistical results in Table 2 showed that during the 0-30d period, the M20 group had a lower body length-specific growth rate compared to the C group, while both the L20 and M10L10 groups had higher rates than the control group. At 30–60 days the M20 group exhibited a higher body length-specific growth rate than the C group, while the L20 and M10L10 groups showed lower rate than the C group. The weight-specific growth rate of M20, L20 and M10L10 groups was higher than that of group C at 0-30d. However, during 30-60d, the body weight-specific growth rate of the M20, L20 and M10L10 groups was lower than that of C group.

**TABLE 2:** Statistics of specific growth rate of body length and body weight n = 30; $\bar{x}$ ±SD.

Group	Time (d)	Body length (cm)	SGR_L_ (%)	Body weight (g)	SGR_W_ (%)
C	0	1.00 ± 0.10	-	0.10 ± 0.01	-
	30	3.90 ± 0.41	4.53	0.75 ± 0.22	6.73
	60	6.25 ± 0.80	1.58	5.30 ± 0.89	6.50
M20	0	1.00 ± 0.10	-	0.10 ± 0.01	-
	30	3.50 ± 0.34	4.17	0.84 ± 0.18	7.08
	60	6.07 ± 0.55	1.84	4.95 ± 0.85	5.93
L20	0	1.00 ± 0.10	-	0.10 ± 0.01	-
	30	4.08 ± 0.29	4.69	0.90 ± 0.16	7.32
	60	6.26 ± 0.74	1.43	5.19 ± 0.90	5.85
M10L10	0	1.00 ± 0.10	-	0.10 ± 0.01	-
	30	3.99 ± 0.36	4.62	0.84 ± 0.18	7.08
	60	6.00 ± 0.60	1.36	4.94 ± 0.82	5.91

SGR_L_: specific growth rate of body length; SGR_W_: specific growth rate of body weight.

### Sex ratio and gonadal histology of pseudo-males

**FIGURE 2:**
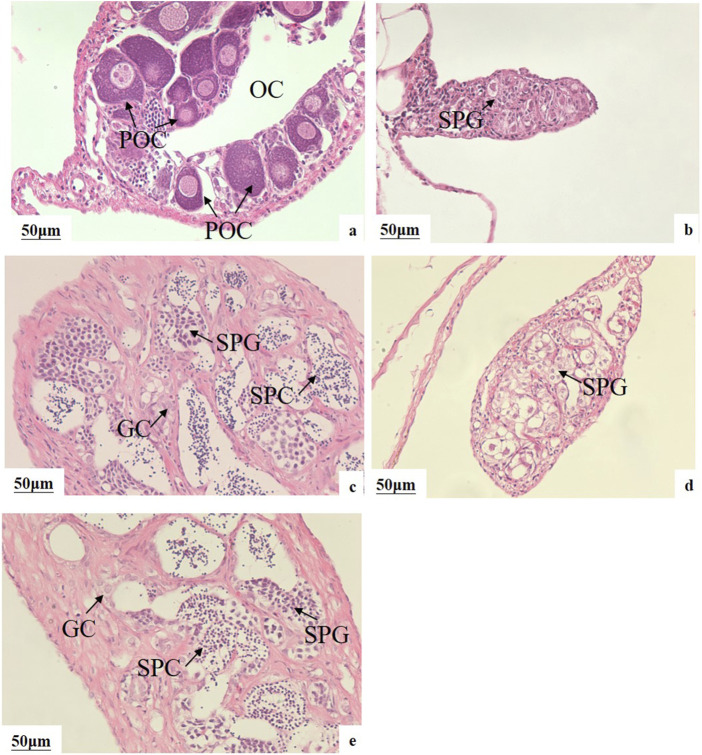
Effects of MT and LE on gonad development of 75-day-old LMB **(A)** female in C group; **(B)** male in C group; **(C)** pseudo-males in M20 group; **(D)** pseudo-males in L20 group; **(E)** pseudo-males in M10L10 group; OC: ovarian cavity; POC: primary oocytes; SPG: spermatogonia; GC: germ cells; SPC: spermatocytes.

The genetic sex of all fish was determined using sex-linked molecular markers, and this information was combined with HE sections to identify pseudo-males. After a 60-day feeding period, the sex reversal ratio in the M20, L20, M10L10 were 95.00%, 80.00%, 76.47%, respectively (Table 3). The control female had ovarian cavity and a great deal of primary oocytes (Figure 2A). Compared to the control male fish (Figure 2B), the gonads of pseudo-male fish in each experimental group exhibited testicular development (Figures 2C–E). Spermatogonium were observed in the L20 group, while no spermatocytes were detected (Figure 2D). Conversely, a significant abundance of spermatogonia and spermatocytes were observed in the M20 and M10L10 groups (Figures 2C,E).

**TABLE 3:** Sex reversal ratio of 75-day-old and12-month-old LMB treated with MT and LE.

Group	Fish examined	75-day-old				12-month-old			
		Male	Female	Pseudo-male	Sex reversal ratio	Male	Female	Pseudo-male	Sex reversal ratio
C	30	14	16	0	0	15	15	0	0
M20	30	10	20	19	95.00%	14	16	16	100.00%
L20	30	15	15	12	80.00%	15	15	13	86.67%
M10L10	30	13	17	13	76.47%	15	15	11	73.33%

**FIGURE 3:**
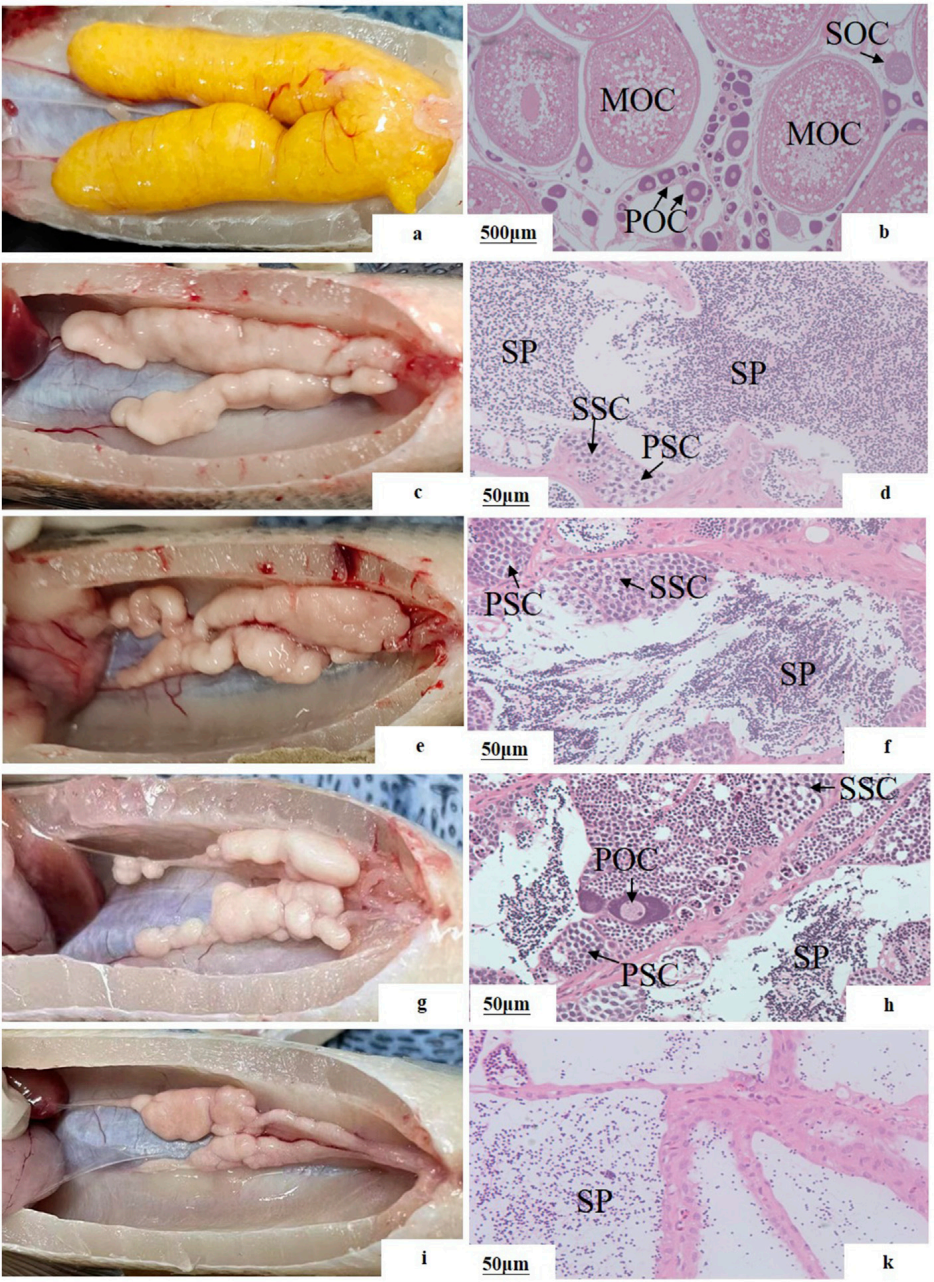
Gonadal structure of 12-month-old pseudo-male LMB **(A, B)** female in C group; **(C, D)** male in C group; **(E, F)** pseudo-males in M20 group; **(G, H)** pseudo-males in L20 group; **(I, J)** pseudo-males in M10L10 group; PSC: primary spermatocytes; SSC: secondary spermatocytes; SP: sperms; POC: primary oocytes; SOC: secondary oocytes; MOC: mature oocytes.

The sex reversal ratio of M20, L20, M10L10 at 12 months were 100.00%, 86.67%, 73.33%, respectively (Table 3). The female in control group (Figures 3A,B) is different to other groups. Compared to the control male fish (Figures 3C,D), the gonad of the pseudo-males in M20 was similar to control group (Figures 3E,F). The gonads of the pseudo-males in the L20 and M10L10 groups had completely developed into *dysmorphic* testes (Figures 3G,I). Histological analysis revealed the presence of oocytes in the gonads of the L20 group (Figure 3H), while the gonads of other groups transformed entirely into testes (Figures 3F,K). In the control group, sperms were most evenly distributed (Figure 3D), followed by the M20 group and then the M10L10 group (Figures 3F,K).

The formular of calculating the ratio are as follows:

$$\frac{N1}{N2}\times 100\%$$

*N1*: the number of pseudo-males in all females; *N2* the number of all females.

### Pair breeding results of 12-month-old pseudo-males

The sex of 200 randomly selected fish from both the all-female group and the control group was observed, and the proportion of females was calculated. The results showed that 92.00% of the offspring in the all-female group were female, while it was 46.50% in the control group (Table. 4).

**TABLE 4:** Female proportion in offspring of pseudo-male LMB in M20.

Group	Fish examined	Female	Male	Female ratio (%)
control group	200	93	107	46.50
all-female group	200	184	16	92.00

### E_2_ and T concentration in serum of 12-month-old pseudo-males

**FIGURE 4:**
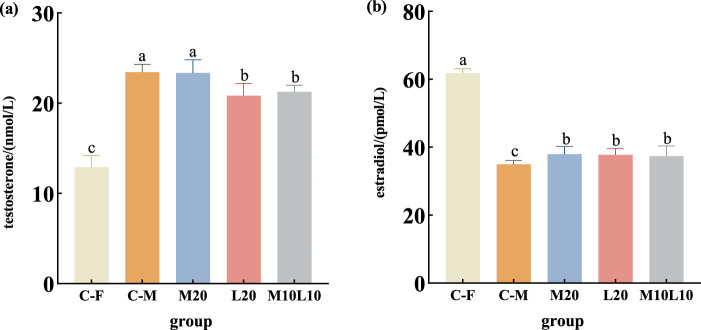
Concentrations of testosterone **(A)** and estradiol **(B)** in 12-month-old pseudo-male LMB. C–F: female in C group; C–M: male in C group; M20: physiological males in M20 group; L20: physiological males in L20 group; M10L10: physiological males in M10L10 group. Note: Different letters between the treatments in the same period show significant difference (*p* < 0.05), the same letter indicates that the difference is not significant (*p* > 0.05).

The levels of E_2_ and T were determined in the pseudo-males from the experimental groups, as well as in the male and female from the control group at 12-month-old (Figure 4). The T content in pseudo-male fish across all experimental groups was significantly higher than that of female fish in C-F group (*p* < 0.05) (Figure 4A). No significant difference existed between male fish in the M20 group and the C-M group (*p* > 0.05) (Figure 4A), whereas both the L20 and the M10L10 groups showed significantly lower compared to those in the C-M group (*p* < 0.05) (Figure 4A). The E_2_ content of pseudo-male fish across all experimental groups was significantly lower than that of females in the C-F group but significantly higher than that of males in the C-M group (*p* < 0.05) (Figure 4B).

### Expression of *cyp19a1a* and *dmrt1* in gonads of 12-month-old pseudo-males

**FIGURE 5:**
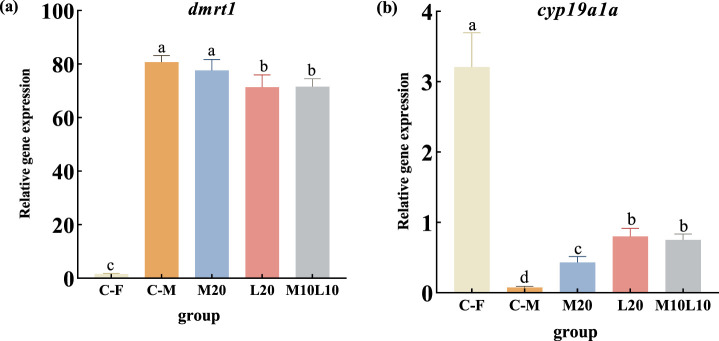
Expression of *dmrt1* **(A)** and *cyp19a1a* **(B)** in 12-month-old pseudo-male LMB. C–F: female in C group; C–M: male in C group; M20: physiological males in M20 group; L20: physiological males in L20 group; M10L10: physiological males in M10L10 group; Note: Different letters between the treatments in the same period show significant difference (*p* < 0.05), the same letter indicates that the difference is not significant (*p* > 0.05).

The mRNA expression of *dmrt1* and *cyp19a1a* were analyzed in gonad tissues of pseudo-males from the experimental group, as well as normal male and female of the control group (C-F group and C-M group) at 12-month-old. Results showed that the expression level of *dmrt1* was significantly higher in both the C-M group and all experimental groups compared to the C-F group (*p* < 0.05) (Figure 5A). Additionally, within the experimental groups, *dmrt1* expression was significantly higher in the M20 group compared to the L20 and M10L10 groups (*p* < 0.05), with no significant difference observed between M20 group and control group regarding *dmrt1* expression (*p* > 0.05) (Figure 5A). In addition, the expression level of *Dmrt1* was significantly lower in L20 and M10L10 groups compared to the C-M group (Figure 5A) The expression level of *cyp19a1a* was significantly lower than that of the C-F group, but higher than that of the C-M group (*p* < 0.05) (Figure 5B). In the M20 group, it showed a significant decrease compared to both the L20 and M10L10 groups (*p* < 0.05) (Figure 5B).

## Discussion

### Effects of MT and LE feeding on the growth of LMB

MT, a highly effective sex reversal inducer, affects fish growth in addition to sex differentiation. Low concentrations of MT can either promote fish growth or have no effect, while high concentrations can inhibit fish growth (Rinchard et al., 1999). The study revealed that the growth of king salmon (*O. tshawytscha*) was unaffected by a 10 mg/kg MT treatment compared to the control group (Hunter et al., 1983). However, administering 20 mg/kg MT significantly enhanced yellow perch (*Perca flavencens*) growth, resulting in a significantly higher body weight in the experimental group compared to the control group (Othman et al., 2022). In the experiment on masculinization induction in LMB, a dosage of 60 mg/kg MT had no effect on its growth (Arslan et al., 2010), but when increased to 100 mg/kg, its significantly inhibited growth (Zhou et al., 2021). However, both dosages of MT supplementation promoted the growth of nile tilapia (*Oreochromis niloticus*) (Shahanoor et al., 2023). It can be observed that different species exhibit varying levels digestion, and absorption adaptability to the same concentration of MT. The effect of LE on fish growth is similar to MT. Qin et al. (2019) compared the effects of feeding different concentrations of LE (1, 10 and 100 mg/kg) on the growth of spotted maigre (*Nibea mitsukurii*). They found that doses of 1 and 10 mg/kg had no impact t on growth, while a dose of 100 mg/kg significantly suppressed it. Shen et al. (2015) reported that feeding yellow catfish with a dose of 20 mg/kg of LE for 60 days at the age of 10 days could promote their growth. In this study, the growth of largemouth bass was not significantly affected in any of treatment groups, consistent with the conclusion that low doses of MT or LE had no discernible effect on fish growth.

### Effects of MT and LE on sex ratio and gonad development of LMB

Among various exogenous male hormones, MT is extensively used to induce pseudo-male fish due to its effective induction capabilities (Pandian and Sheela, 1995). For the same species, when using identical induction agents and durations, the determining factor for the induction effect of MT was found to be initiation time rather than concentration. Porter et al. (1996) fed a dosage of 60 mg/kg MT to feed 40-day-old LMB, resulting in a male ratio of 52.4% after 60 days. However, Garrett et al. (1989) obtained a male ratio of 90.00% with the same treatment duration by reducing the MT concentration to 50 mg/kg and initiating feeding at an earlier age (35 days old). The timing of Garrett’s hormone administration, which was closer to the period of sexual differentiation in LMB (20–30 days old) (Du et al., 2022), may explain this difference. In this study, a lower concentration of MT (20 mg/kg) was fed to 15dph LMB for 60 days, and had 95.00% sex reversal ratio. These results revealed that the accurate time of fish sex differentiation is crucial for improving induction efficiency.

MT directly participates in the induction process of masculinization, while LE indirectly affects the direction of gonadal differentiation by inhibiting aromatase activity. Although their mechanisms are different, both can induce masculinization in genetic female fish (Liu et al., 2019). The male ratio in tilapia reached 98.50% after 60 days of being fed with 200 mg/kg LE, (Singh, 2013). Similar to our current observation, in yellow catfish, after 60 days of being fed with 20 mg/kg LE, the male ratio was 75.50% (Shen et al., 2015). In this study, the sex reversal ratio fed the same dose of LE was 80.00%, while the sex reversal rate in the M10L10 group was only 76.47%. This could be attributed to the abrupt decrease in estrogen levels and increase in androgen activity caused by LE-induced inhibition of aromatase activity, which causes estrogen metabolism compensation and ultimately impacts masculinized effectiveness (Hornung et al., 2004). Furthermore, when comparing the sex proportion between two time points, it is more likely that differences in sample numbers account for the variations observed after 12 months, suggesting that inducing masculinization during the undifferentiated stage of gonad development and sustaining it for a sufficient time can completely change the sex of the genetic females to males (Yamamoto, 1969).

Moreover, hormone-induced sexual reversal in teleost often alters the process of the gonadal development, resulting in delayed or accelerated gonadal development and maturation. After feeding 30-day-old catfish (*Clarias fuscus*) with 100 and 200 μg/L MT, the development of testes was found to be 3–5 days earlier (Li et al., 2013). Similarly, yellow catfish fed with 20 mg/kg MT exhibited significantly faster spermatogenesis compared to the control group (Yao, 2007). Spermatic differentiation retardation was observed in pseudo-male LMB when they were fed high concentrations of MT (100 mg/kg). The testis structure exhibited predominantly interstitial tissue, with a reduced number of spermatocytes (Zhou et al., 2021). In this study, the testes development of treatment group (excluding the L20 group) showed accelerated growth compared to the control group, indicating that a high dose of MT not only inhibits growth but also impedes testis development, while lower dose promotes it.

### Gonadal development of 12-month-old LMB in pseudo-males

MT has been used to obtain pseudo-males in species such as zebrafish (*Danio rerio*) (Matthews et al., 2022), cherry burb (*Barbus titteya*) (Yu, 2012), and nile tilapia (Shahanoor et al., 2023). Although pseudo-male fish were easily obtained through MT treatment, there was a higher possibility of abnormal testes development (Pandian and Sheela, 1995). Ankley et al. (2001) found that the addition of MT above 100 mg/L induced necrosis in spermatocytes and atrophy in testes of fathead minnow (*Pimephales Promelas*). Johnstone et al. (1979) reported the absence of ductus deferens during masculinization induction in female rainbow trout, but sperm production was still observed. The dusky grouper (*Epinephelus Marginatus*) treated with MT has smaller testes and delayed development (Sarter et al., 2006), which is consistent with this study. However, successful fertilization with female fish was observed in the pseudo-male fish in the 20 mg/kg MT group.

Treatment with aromatase inhibitors can completely induce genetic sex change in female protogynous wrasse (*Halichoeres trimaculatus*) within a short period of time (Ryo et al., 2009). Facultative gonads were observed in yellow perch after cessation of LE feeding (Othman et al., 2022). However, in studies on pseudo-male yellow catfish, only poor testes without sperm lobules were obtained with LE treatment (Yang et al., 2018). In the L20 group of this experiment, ovotestis gonads were observed, whereas no such gonads were observed in the other treatment groups. It is possible that the concentration of LE provided in this study was insufficient to achieve a satisfactory induction effect. Additionally, throughout the developmental process, a small number of germ cells underwent differentiation into ovarian cells under the regulation of genetic mechanisms. In the M10L10 treatment group, large cavities were observed simultaneously, possibly due to the inability of mature sperms to be discharged and resulting in sperm degradation (Shen et al., 2015).

### Concentration of sex steroid hormones and expression of sex-related genes in 12-month-old pseudo-male LMB

Sex steroid hormones, namely, estrogen and androgen, play a crucial physiological regulatory role in the reproduction and development of teleost (Yao et al., 2019). Estrogen, mainly refers to estradiol, primarily functions in ovarian differentiation and oocyte maturation, while androgen, mainly testosterone, participates in spermatogenesis and testes differentiation (Jiang, 2014). Additionally, the *cyp19a1a* and *dmrt1* genes played a significant role in gonadal development and sexual differentiation, contributing to the differentiation and maturation of ovaries and spermatozoa, respectively. They were often used as distinctive markers for fish developing into either female or male phenotypes (Devlin and Nagahama, 2002; Matson et al., 2011). During the breeding period, in normal male fish, when *dmrt1* expression reached its peak, the serum T content also sharply increased, meanwhile, *cyp19a1a* expression was lowest, the E_2_ content also decreased significantly (Yamada et al., 2002). In contrast, normal female fish exhibited opposite changes (Adolfi et al., 2015). In this study, the content of testosterone in serum of each treatment group was consistent with the expression level of *dmrt1*, and the level of estradiol was corresponded to the expression level of aromatase. These indicated that the profile of steroids (T and E_2_) and genes (*cyp19a1a* and *dmrt1)* was similar between pseudo-males and normal males. However, sex steroid hormones levels and gonadal development-related gene expression levels in pseudo-males were lower than those in the C-M group. This may be one reason why the maturation of LMB’s testes was hypoplasia in the L20 and M10L10 groups. In subsequent breeding seasons, we can try to enhance male hormone levels and promote testes development by supplementing exogenous hormones.

## Conclusion

The treatment of 20 mg/kg MT, 20 mg/kg LE and 10 mg/kg MT+10 mg/kg LE on 15-day-old LMB for 60 days did significantly affect their growth. The male proportion was 95.00%, 80.00% and 76.47%, respectively. At the age of 12 months, L20 and M10L10 groups exhibited *dysmorphia* testes, while the pseudo-males in the group treated with 20 mg/kg MT had testicular morphology most similar to normal males. Additionally, the testosterone content and *dmrt1* expression in pseudo-males were extremely similar to those in normal males. The pseudo-male from this group was selected for breeding with a normal female fish, resulting in a female proportion of their offspring at 92.00%.

## Data Availability

The raw data supporting the conclusions of this article will be made available by the authors, without undue reservation.
